# INFORMAL CAREGIVING NETWORKS FOR PERSONS WITH DEMENTIA SUPERIMPOSED ON COMPLEX MULTIMORBIDITY

**DOI:** 10.1093/geroni/igac059.2098

**Published:** 2022-12-20

**Authors:** Mi-Kyung Song, Sudeshna Paul, Mary Beth Happ

**Affiliations:** Emory University, Atlanta, Georgia, United States; Emory University, Atlanta, Georgia, United States; The Ohio State University, Columbus, Ohio, United States

## Abstract

Informal caregiving research has focused on the primary caregiver and caregiver-patient dyad. Thus, we know little about caregiving beyond the dyadic relationship. This study was to gain a comprehensive understanding of informal caregiving networks for individuals with dementia superimposed on complex multimorbidity. We used egocentric social network analysis to obtain caregiving information of 46 patients with moderate to severe cognitive impairment, 5 chronic conditions on average, and undergoing hemodialysis (4.3 mean years). Most patients (n=35, 77.8%) were Black, 22 (47.8%) male, and mean age of 73.9 years. Starting with the primary family caregiver (FCG), up to 2 additional FCGs were recruited for each patient, totaling 76 FCGs (46 primary, 30 non-primary). Most were a child of the patient (n=39, 51.3%), female (n=57, 75%), and 54.2 years of age. Of the 46 networks, 16 (35%) included only one FCG (singletons). Multimember networks (n=30, 65%) provided longer caregiving than singletons (7.7 vs 3.8 years, p=0.008). Average network size was 2.8, and 26 (54.5%) networks had at least one male caregiver. Among the 30 multimember networks, average size was 3.8, density (proportion of possible ties) was 0.9, and mean degree and maximum degree (number of ties per member to other network members) were 2.5 and 2.8, respectively. Higher mean and maximum degrees were associated with fewer 12-month patient hospitalizations (r=-0.47, p=0.01; r=-0.43, p=0.02, respectively). Including additional caregiver informants significantly increased network size, ties and maximum degree centrality compared to those based on primary caregiver only, allowing for fuller network description.

